# Comparative transcriptome analysis provides insights into the gene regulation network of cytoplasmic male sterility in chilli pepper

**DOI:** 10.1093/aobpla/plaf070

**Published:** 2026-01-07

**Authors:** Meng Wang, Hu Zhao, Xing Wu, Zongjun Li, Zengjing Zhao, Mingxia Gong, Liping Huang, Risheng Wang

**Affiliations:** Vegetable Research Institute of Guangxi Zhuang Autonomous Region Academy of Agricultural Sciences, Guangxi Vegetable Breeding and New Technology Research Laboratory, 174 University East Road, Nanning 530007, Guangxi, P.R. China; Vegetable Research Institute of Guangxi Zhuang Autonomous Region Academy of Agricultural Sciences, Guangxi Vegetable Breeding and New Technology Research Laboratory, 174 University East Road, Nanning 530007, Guangxi, P.R. China; Vegetable Research Institute of Guangxi Zhuang Autonomous Region Academy of Agricultural Sciences, Guangxi Vegetable Breeding and New Technology Research Laboratory, 174 University East Road, Nanning 530007, Guangxi, P.R. China; Vegetable Research Institute of Guangxi Zhuang Autonomous Region Academy of Agricultural Sciences, Guangxi Vegetable Breeding and New Technology Research Laboratory, 174 University East Road, Nanning 530007, Guangxi, P.R. China; Vegetable Research Institute of Guangxi Zhuang Autonomous Region Academy of Agricultural Sciences, Guangxi Vegetable Breeding and New Technology Research Laboratory, 174 University East Road, Nanning 530007, Guangxi, P.R. China; Vegetable Research Institute of Guangxi Zhuang Autonomous Region Academy of Agricultural Sciences, Guangxi Vegetable Breeding and New Technology Research Laboratory, 174 University East Road, Nanning 530007, Guangxi, P.R. China; Vegetable Research Institute of Guangxi Zhuang Autonomous Region Academy of Agricultural Sciences, Guangxi Vegetable Breeding and New Technology Research Laboratory, 174 University East Road, Nanning 530007, Guangxi, P.R. China; Vegetable Research Institute of Guangxi Zhuang Autonomous Region Academy of Agricultural Sciences, Guangxi Vegetable Breeding and New Technology Research Laboratory, 174 University East Road, Nanning 530007, Guangxi, P.R. China; Molecular Function & Environment

**Keywords:** chilli pepper, fertility, sterility, restorer-of-fertility genes, regulatory network, developmental stages, lines, differentially expressed genes, transcriptomic analysis, CMS/*Rf* system

## Abstract

Cytoplasmic male sterility (CMS) is a common biological phenomenon in chilli pepper hybrid production. Although several restorer-of-fertility (*Rf*) genes have been identified in pepper CMS lines, a regulatory network has yet to be constructed. Morphological characteristics of the sterile, maintainer, and restorer flower buds were studied at three different developmental stages. We conducted transcriptome analysis of the CMS/*Rf* system in pepper plants. Pentose and glucuronate interconversion pathways were particularly enriched in most comparison groups. In addition, differentially expressed genes among the different lines at flower bud stages 2 and 3 were generally enriched in amino sugar and nucleotide sugar metabolism pathways. In our study, the small auxin upregulated RNA (*SAUR*), *A-ARR* and *GH3* genes in the plant hormone signal transduction pathway, *Capana12g000348*, *CKX7* and *cis*-zeatin O-glucosyltransferase (*CISZOG*) genes in the zeatin biosynthesis pathway, and receptor-like protein kinase 2 (*RLK2*) in the germplasm development signal pathway showed gradual upregulation across developmental stages in the restorer line. However, expression of these genes was stable in the sterile and maintainer lines. qRT-PCR analysis showed that *SAUR*, *A-ARR*, *GH3, Capana12g000348*, *CKX7*, *CISZOG*, *CRE1, AHP* and *TIR1* participate in CMS fertility regulation in chilli pepper. We constructed a regulatory network based on critical genes. Overall, our research provides a solid theoretical foundation for the development of CMS fertility studies on chilli pepper.

## Teaser

This study investigates cytoplasmic male sterility (CMS) in chilli pepper by comparing gene expression in sterile, maintainer, and restorer lines across different bud developmental stages. Key genes involved in hormone signaling and sugar metabolism were identified as crucial for fertility restoration. The findings establish a regulatory network for CMS, offering valuable insights for improving hybrid breeding efficiency and advancing molecular studies on male sterility in peppers.

## Introduction

The genus *Capsicum* comprises over 30 species, including five crop species, namely *C. annuum*, *C. baccatum*, *C. chinense*, *C. frutescens* and *C. pubescens*, of which *C. annuum* is the most widely cultivated (Ma et al. 2024). *Capsicum* spp. are the largest culinary spice crops and the third largest vegetable crops worldwide. Moreover, they are the largest vegetable and condiment crop in China, with the largest planting area, the most diverse processing methods and uses (Nie et al. 2022). The advantages of hybrid chilli peppers are evident, and hybrids account for more than 80% of the pepper varieties used in production (Kim et al. 2006). Currently, hybrid pepper production relies mainly on manual emasculation, which results in high seed production costs and a lack of market competitiveness. Researchers first reported cytoplasmic–nuclear interaction-type male sterility in pepper in 1958, following which breeders bred numerous sterile pepper lines using cytoplasmic male sterility (CMS) lines (Peterson 1958, Xu et al. 2022). Currently, three-line chilli pepper hybrid seed production using CMS is a major trend in chilli pepper breeding (Sun et al. 2017). The mitochondrial sterility and nuclear restoration genes regulate CMS. Applying CMS to hybrid seed production requires male-sterile lines (*rfrf*), male-maintainer lines (*rfrf*) and male-fertility-restoration lines (*RfRf*), which are collectively referred to as triple-line systems (Jo et al. 2010). Restoration lines containing fertility restoration genes (*Rf*) are required for three-line varieties to ensure the fertility of *F*_1_ hybrids. The selection of recovery lines is an important breeding objective in the CMS/*Rf* system. Male sterility genes in the cytoplasm can be obtained through maternal inheritance. Therefore, fertility restoration by nuclear genes is paramount to the CMS/*Rf* system as well as a focal point and ongoing challenge in research (Nie et al. 2022).

The results of previous studies on restorer-of-fertility (*Rf*) genes in pepper are controversial. Most studies have concluded that *Rf* genes in pepper are controlled by a single dominant gene (Jo et al. 2016). In contrast, others believe that it is associated with one main QTL and four minor QTLs linked to the recovery of fertility in pepper (Wang et al. 2004) or comprises two main additive-dominant epistatic genes and one additive-dominant polygenic gene (Wei et al. 2020). In addition, the CMS phenotype was temporarily restored at low temperatures, suggesting that temperature affects the expression of fertility-modifying genes (Min et al. 2008). After the release of the pepper genome sequence (Zhang et al. 2021), several researchers have constructed genetic maps (Zhang et al. 2020) and identified multiple genes with different mechanisms of action related to restoring pepper fertility, including *PPR6* (Jo et al. 2016), *CaRf032* (Zhang et al. 2022) and *Capana06g003028* (Cheng et al. 2020). Long non-coding RNAs (lncRNAs) may also be involved in CMS (Ortega et al. 2020). In summary, the genetic control of chilli pepper fertility is diverse and complex. The chilli pepper genome contains multiple candidate restoration genes (Bartoszewski et al. 2012), and different restoration lines may have genotype-specific restoration genes (Wang et al. 2023).

Transcriptomic analysis has been widely used in studies of CMS fertility in plants. For example, comparative transcriptome profiling of the CMS-D2 and CMS-D8 systems revealed a fertility restoration gene network in upland cotton (Song et al. 2023). Similarly, a comparative transcriptome analysis revealed that tricarboxylic acid cycle-related genes were associated with maize CMS-C fertility restoration (Liu et al. 2018). Transcriptomic analysis has provided novel insights into the mechanism of CMS in tobacco (*Nicotiana tabacum* L.) (Mo et al. 2023) and revealed that heat-responsive miRNAs play a regulatory role in the pollen fertility stability of CMS-D2 restorer lines under high-temperature stress (Zhang et al. 2023). In this study, we compared the transcriptomes of chilli pepper flower buds in sterile, maintainer and restorer lines. The weighted gene co-expression network analysis provided novel insights into the gene regulatory network of chilli pepper plants. A strong positive correlation was observed between the RNA-Seq data and qRT-PCR validation results for *SAUR*, *A-ARR*, *GH3*, *Capana12g000348*, *CKX7*, *CISZOG*, *CRE1, AHP* and *TIR1*. Our results provide a solid theoretical basis for molecular biology studies on CMS fertility in chilli pepper.

## Materials and methods

### Plant material and sampling

The experimental materials used in this study were the self-developed sterile line 014A of chilli pepper, maintainer line 014B and restorer line 014C developed in 2018 by the pepper research team at the Vegetable Research Institute of the Guangxi Academy of Agricultural Sciences. Samples of flower buds from the sterile, maintainer and restorer lines were collected at three developmental stages (Stages 1, 2 and 3). Each line at each stage was sampled in triplicate for subsequent transcriptome sequencing. The varieties used in this study were preserved in the Germplasm Resource Bank of the Vegetable Research Institute, Guangxi Academy of Agricultural Sciences (accession numbers: SC030112031, SC030112032 and SC030113077).

### RNA extraction and RNA-Seq

Total RNA was extracted using the HiPure Total RNA Mini Kit (Magen, Guangzhou, China). Polyadenylated eukaryotic mRNA was enriched using magnetic beads with oligo (dT) and then fragmented via ultrasonication. Double-stranded cDNA was synthesized using fragmented mRNA as the template and random oligonucleotides as primers. Purified double-stranded cDNA was subjected to end repair, A-tailing and adapter ligation. Subsequently, cDNA fragments of approximately 200 bp were selected using AMPure XP beads, followed by PCR amplification and a second purification step using AMPure XP beads to obtain the final library. The integrity and purity of the RNA samples were assessed by agarose gel electrophoresis and spectrophotometry (OD260/280 and OD260/230, respectively). RNA concentration was accurately quantified using a Qubit 2.0 Fluorometer, and RNA integrity was precisely determined using an Agilent 2100 bioanalyzer. Qualified RNA samples were used for library construction and sequencing using the DNBSEQ-T7 platform (MGI Tech., Shenzhen, China) in the PE150 mode, with three biological replicates for each developmental stage of the sterile, maintainer and restorer lines.

### Transcriptome analysis

Raw reads were subjected to quality control using Fastp (Chen et al. 2018). The clean reads were aligned to the reference genome (https://solgenomics.net/organism/Capsicum_annuum/genome) using the HISAT2 software (Kim et al. 2015). Transcripts were reconstructed using StringTie (Shumate et al. 2022), and the expression levels of all genes in each sample were calculated using RSEM (Li and Dewey 2011). Intergroup differential expression analysis was performed using DESeq2 software (Love et al. 2014), with significant DEGs selected based on the following criteria: false discovery rate (FDR) < 0.05, |log2FC|>1. Functional annotation of genes was performed using eggnog mapper (Jaime et al. 2016) for Gene Ontology (GO) (Ashburner et al. 2000) and KEGG annotation (Kanehisa and Goto 2000), and enrichment analysis of the DEGs was performed based on hypergeometric testing.

### Homology identification of germplasm development-related genes

Protein sequences of *Arabidopsis* germplasm development-related genes were retrieved from The *Arabidopsis* Information Resource ((TAIR, https://www.arabidopsis.org/) using the keyword ‘germplasm’). A tBLASTn search was performed against the pepper genome assembly (version 2.15.0) using *Arabidopsis* protein sequences as queries to identify orthologous genes in chilli pepper. The following thresholds were applied to filter significant hits: *E*-value ≤ 1 × 10⁻⁵, Sequence Identity ≥ 30% and Query Coverage ≥ 50%.

### Temporal analysis

Trend analysis of DEGs was conducted using Short Time-series Expression Miner software with the parameters -pro 20 -ratio 1.0. Profiles with *P* < .05 were defined as significant profiles (Ernst and Bar-Joseph 2006). Genes within each significant trend were subjected to GO/KEGG enrichment analysis using the hypergeometric test, and GO terms/KEGG pathways with *P* ≤ .05 were defined as significantly enriched.

### Weighted-correlation network analysis

WGCNA was used to analyse gene co-expression with the power parameter set at 12 (Zhang and Horvath 2005, Langfelder and Horvath 2008). A gene clustering tree was constructed based on the correlation of gene expression, and the gene modules were divided based on the clustering relationships between the genes (minimum module size of 50). After preliminary module division using the Dynamic Tree Cut, highly similar modules were merged based on the similarity of the module eigengene values (similarity threshold = 0.7), resulting in the final division of the modules and merged dynamics.

Traits were defined as stage, line and fertility (fertility status), which was recorded as a binary variable: 0 for sterility and 1 for fertility. Pearson’s correlation coefficients between modules and traits were calculated. Modules that were significantly correlated with traits were further subjected to GO/KEGG enrichment analysis based on a hypergeometric test.

### qRT-PCR validation

Isolated total RNA was reverse-transcribed using MonScript^TM^ 5× RTIII All-in-One Mix with dsDNase (Monad Biotech Co., Ltd., China) following the manufacturer’s instructions. qRT-PCR was performed using the SYBR Green PCR Master Mix (Qiagen, Hilden, Germany). Three replicates were performed for each sample group, and the 2^−ΔΔCt^ method was used for data analysis. All the primer sequences used in this study are listed in Supplementary Table S7. The correlation between the RNA-Seq and qRT-PCR results was assessed by calculating the Pearson correlation coefficients.

### Statistical analysis

Genes with similar trends in lines A and B but different trends in line C were defined as key trend genes. Fisher’s exact test was used to enrich WGCNA modules by comparing the number of key trend genes and total genes in the modules, and modules with statistical significance set at *P* ≤ .05.

## Results

### Stable CMS/*Rf* system in pepper

**Figure 1:**
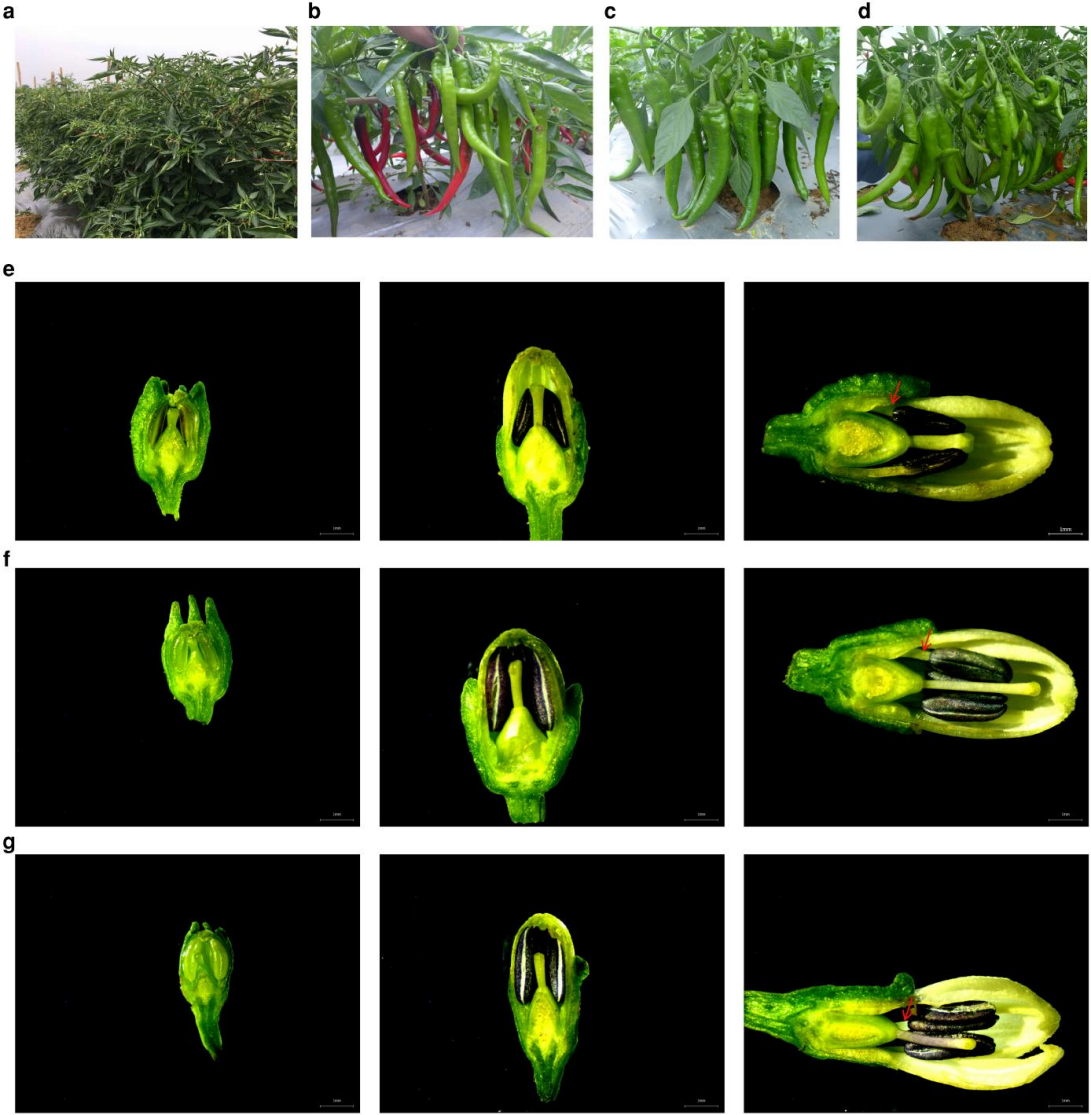
Photographs of the sterile line 014A (a), maintainer line 014B (b), restorer line 014C (c), and the hybrid 014A × 014C (d), and microscopic morphology of flower buds from the sterile line (e), maintainer line (f) and restorer line (g) at three different developmental stages, corresponding (from left to right) to stages 1, 2, and 3.

The sterile line 014A exhibited strong cross-compatibility and a high fruit-set rate (Fig. 1a). Maintainer line 014B is an advanced inbred line developed through selective breeding and is characterized by a high fruit-set rate and strong disease resistance (Fig. 1b). The restorer line, 014C, was selected from over 500 advanced-generation self-crossed lines with excellent traits. It is a medium-sized, high-yielding, cow-horn pepper with strong, continuous fruit-setting ability and high disease resistance (Fig. 1c). This is highly compatible with the sterile line 014A. The *F*_1_ hybrid exhibited normal pollen, good fruit set and a strong restoration ability (Fig. 1d).

We observed the morphological characteristics of the flower buds of the sterile, maintainer and restorer lines during the three developmental stages (Fig. 1e–g). The results showed that the sterile line exhibited short filaments (red arrow in Fig. 1e), a relatively short pistil, and completely sterile, shrivelled anthers (Fig. 1e). The maintainer line was isogenic to the sterile line, but produced fertile pollen (Fig. 1f and Supplementary Figure S1). The restorer line appeared distinct from both the sterile and maintainer lines, and contained normal anthers (Fig. 1g). Overall, the anthers of the sterile line were relatively small and underdeveloped. In contrast, those of both the maintainer and restorer lines were large and plump (Fig. 1e–g). Notably, in the sterile line, no normal pollen was observed at Stage 1 (the smallest buds with petals concealed within the calyx), any pollen present was minimal in quantity and abnormal in shape. In contrast, almost no pollen was observed at Stages 2 (medium-sized buds with petals exposed, light green, but the length of the exposed petals was less than the length of the calyx) and 3 (buds near maturity with white petals exposed, the length of which was greater than the length of the calyx) (Supplementary Figure S1). In contrast, the maintainer and restorer lines exhibited tetrads in Stage 1, uninucleated microspores with rough cell walls, and a large clustered nucleus in Stage 2, and nearly mature binucleate pollen with a spherical shape and clearly visible germination pores in Stage 3 (Supplementary Figure S1).

### Transcriptome analysis of CMS/*Rf* system in pepper

**Figure 2:**
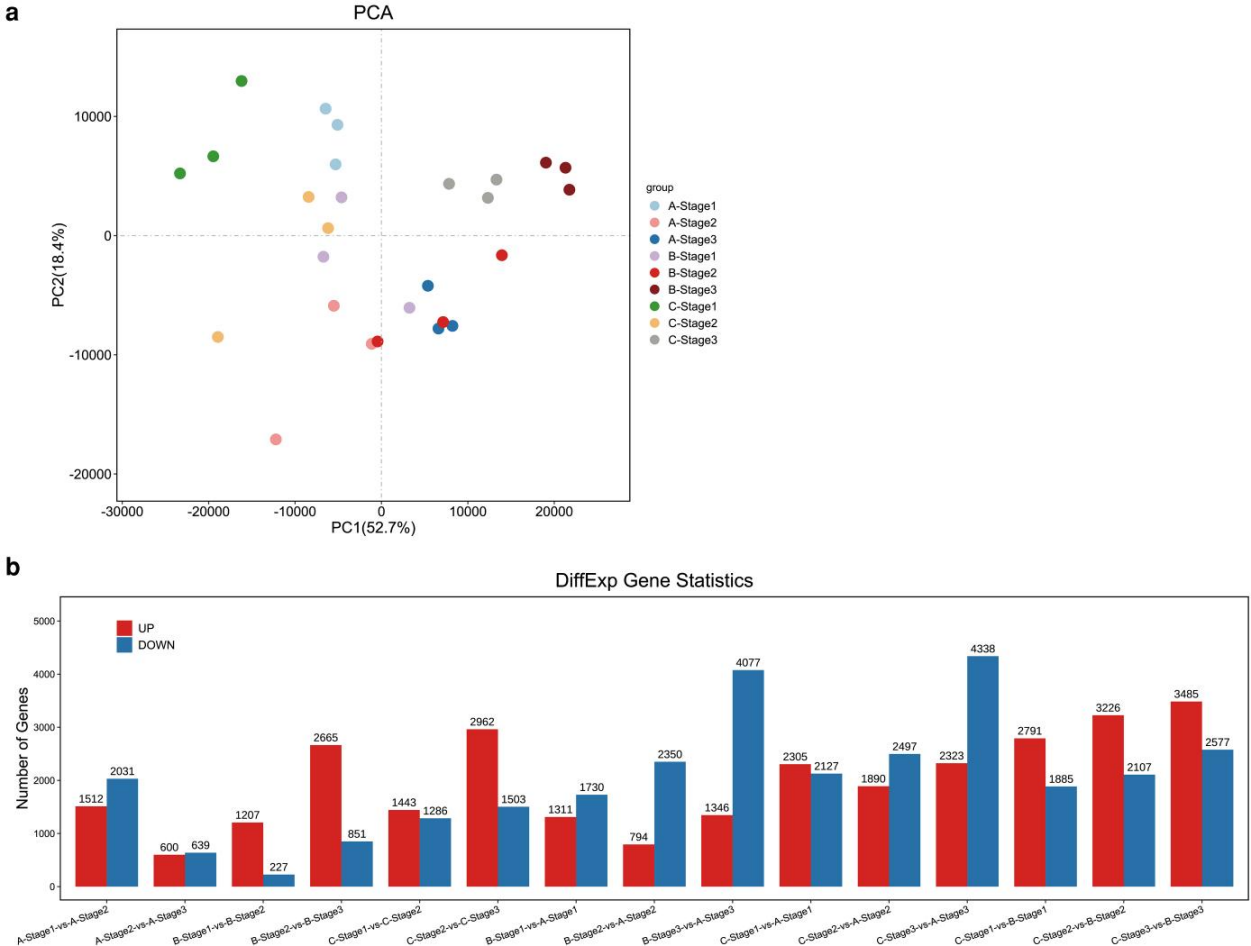
Transcriptome PCA analysis and differential analysis. (a) PCA scatter plot. (b) Bar chart of the number of DEGs. A, B and C represent sterile line, maintainer line and restorer line, respectively.

We further performed transcriptome sequencing of the flower buds of the sterile, maintainer and restorer lines at three different developmental stages, obtaining an average of 6.14 Gb raw data per sample and yielding an average of 6.12 Gb clean data per sample after quality control (Supplementary Table S1). The principal component analysis (PCA) results indicated that samples within the same line tended to cluster, implying a high degree of consistency among samples of the same line. In contrast, samples from different lines were more distant, suggesting significant differences between them (Fig. 2a).

Differential expression analysis was conducted on samples from the three lines (designated A [sterile line 014A], B [maintainer line 014B] and C [restorer line 014C]) at the same stage and on samples from each line at adjacent stages, including Stages 1, 2 and 3 (Fig. 2b and Supplementary Table S2). The comparisons of the highest number of differentially expressed genes (DEGs) were C-Stage3-vs-A-Stage3 (line C vs. line A at Stage 3), B-Stage3-vs-A-Stage3 (line B vs. line A at Stage 3) and C-Stage3-vs-B-Stage3 (line C vs. line B at Stage 3). The number of DEGs in Stage 3 was generally higher than those in Stages 1 and 2, indicating that more genes were mobilized to participate in regulating pepper fertility as flower buds matured (Fig. 2b and Supplementary Table S2).

**Figure 3:**
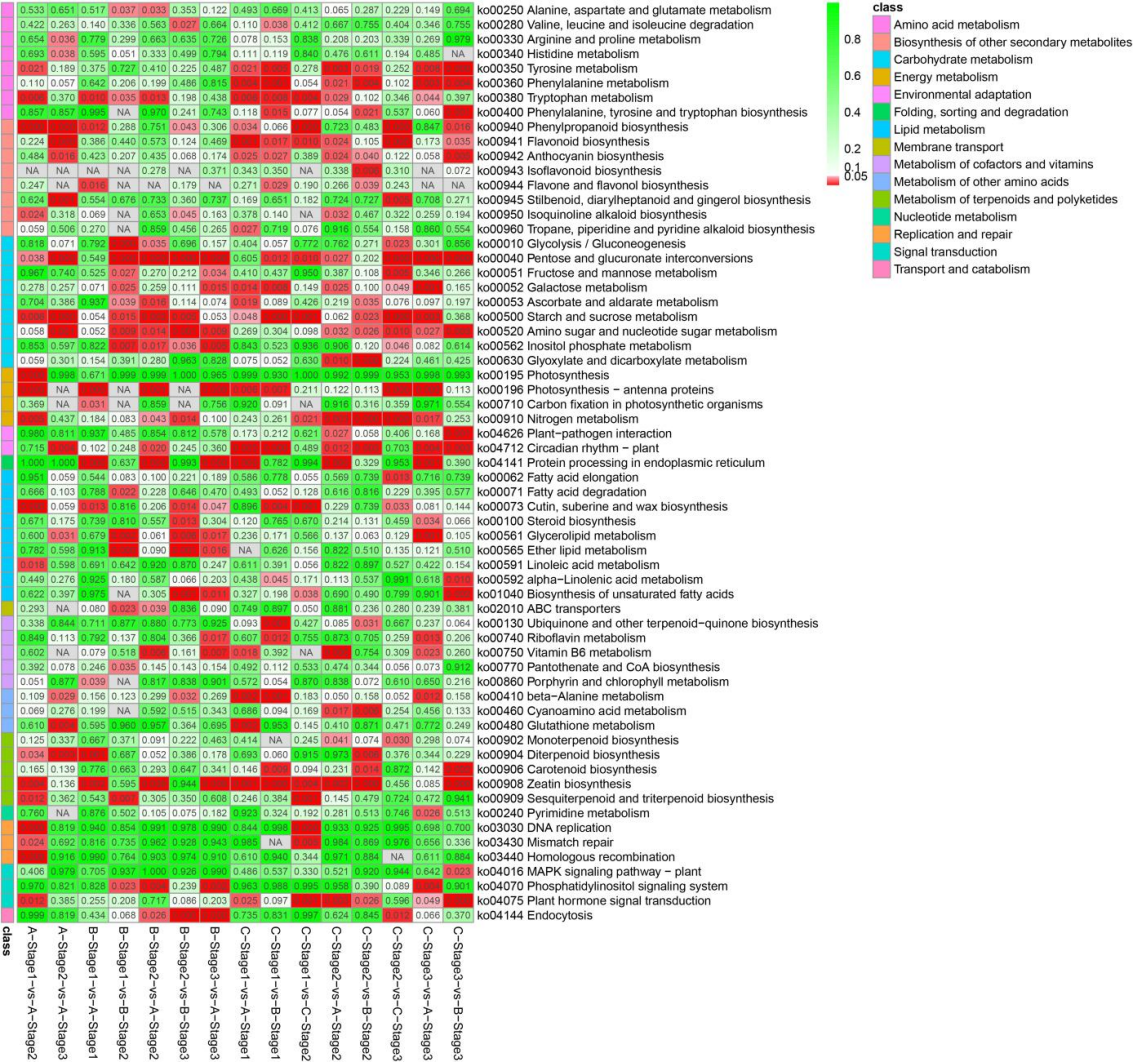
KEGG pathway enrichment analysis of DEGs, with numbers in the heatmap indicating the *P* values of the enrichment analysis, where red and green represent *P* ≤ .05 and *P* > .05, respectively. A, B and C represent the sterile, maintainer and restorer lines, respectively.

Kyoto Encyclopedia of Genes and Genomes (KEGG) pathway enrichment analysis showed that the DEGs in the sterile line between adjacent stages were enriched in phenylpropanoid biosynthesis, pentose and glucuronate interconversions, starch and sucrose metabolism and diterpenoid biosynthesis pathways (Fig. 3). DEGs between adjacent stages in the maintainer line were enriched in pentose and glucuronate interconversion, starch and sucrose metabolism, amino sugar and nucleotide sugar metabolism, inositol phosphate metabolism, glycerolipid metabolism and ether lipid metabolism (Fig. 3). DEGs between adjacent stages in the restorer line were enriched in phenylpropanoid biosynthesis; flavonoid biosynthesis; pentose and glucuronate interconversion; starch and sucrose metabolism; nitrogen metabolism; and cutin, suberin, and wax biosynthesis pathways (Fig. 3). Among the three lines, DEGs were enriched in tryptophan metabolism, photosynthesis-antenna proteins and zeatin biosynthesis at Stage 1 (Fig. 3); amino sugar and nucleotide sugar metabolism, nitrogen metabolism, plant circadian rhythm-plant and zeatin biosynthesis at Stage 2 (Fig. 3); and pentose and glucuronate interconversions and amino sugar and nucleotide sugar metabolism at Stage 3 (Fig. 3). Pentose and glucuronate interconversion pathways were particularly enriched in most comparison groups (Fig. 3). In addition, DEGs among the three different lines at flower bud Stages 2 and 3 were generally enriched in amino sugar and nucleotide sugar metabolism pathways (Fig. 3).

### Comparison of trends in the pepper CMS/*Rf* system

**Figure 4:**
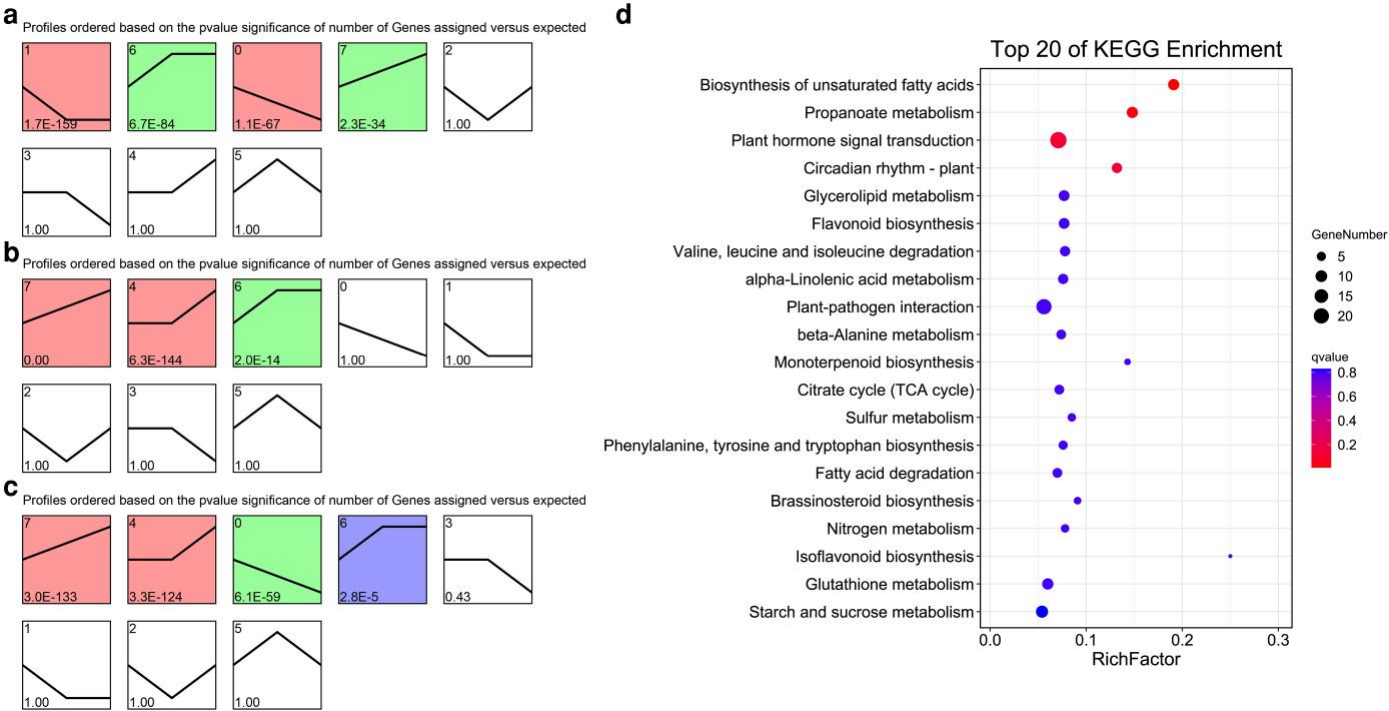
Trend analysis and functional enrichment analysis of the significant profiles. (a-c) Trend analysis for the sterile line (Trend_A), the maintainer line (Trend_B) and the restorer line (Trend_C), respectively, with *P* values indicated in the lower left corner of the profile plots, and significant profiles highlighted with coloured backgrounds. (d) KEGG enrichment analysis for genes with similar trends between Trend_A and Trend_B, but different from Trend_C.

We conducted a trend analysis of the DEGs in samples from the sterile, maintainer and restorer lines according to their developmental stages. Notably, the significant enrichment trends included profiles 0, 1, 6 and 7 for the sterile line samples (Fig. 4a); profiles 4, 6 and 7 for the maintainer line samples (Fig. 4b); and profiles 0, 4, 6 and 7 for the restorer line samples (Fig. 4c).

To identify high-confidence CMS-associated genes while minimizing noise from nuclear background variation, we identified genes with similar trends in Trend_A (sterile line) and Trend_B (maintainer line) but a different trend in Trend_C (restorer line) as key trend genes and performed an enrichment analysis (Fig. 4d, Supplementary Figure S1 and Supplementary Table S3). The results indicated that these genes were primarily involved in DNA-binding transcription factor, naringenin 3-dioxygenase, transcription regulator, GMP synthase, acyl-CoA dehydrogenase, flavanone 7-O-beta-glucosyltransferase, acyl-CoA oxidase and oxidoreductase activities, *etc* (Supplementary Figure S2). Additionally, these genes were involved in the biosynthesis of unsaturated fatty acids, propanoate metabolism, plant hormone signal transduction and plant circadian rhythm pathways (Fig. 4d).

### Weighted gene co-expression network analysis in the pepper CMS/*Rf* system

**Figure 5:**
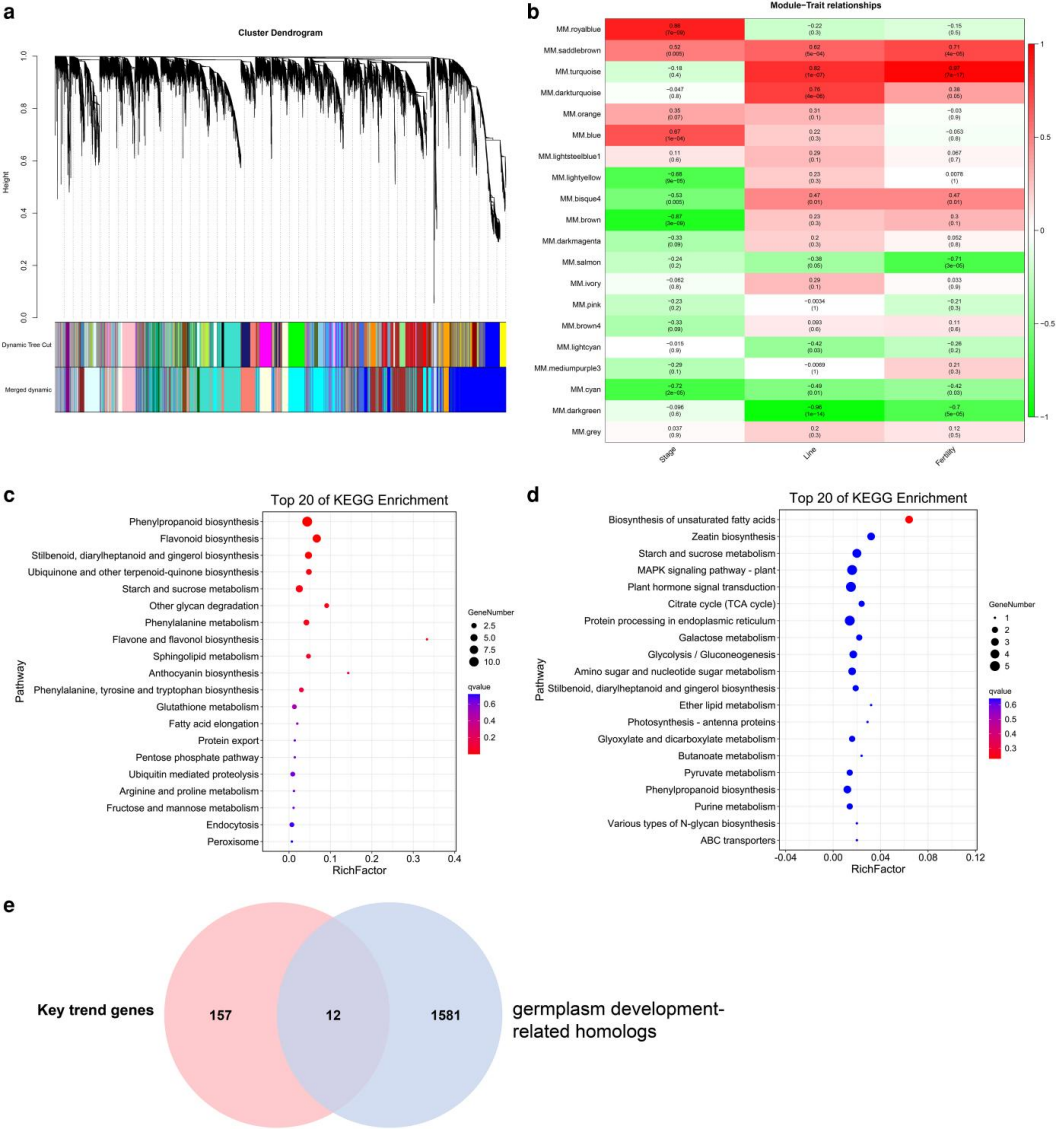
WGCNA analysis and module gene enrichment analysis. (a) WGCNA module tree. Dynamic Tree Cut represents the module division based on clustering results; Merged dynamic represents the final module division after merging modules with similar expression patterns based on module similarity. (b) Modules-trait correlation heatmap. Stage, Line and Fertility indicate different developmental stages, lines and fertility status, respectively. (c, d) KEGG enrichment analysis of genes in darkmagenta (c) and saddlebrown (d) modules. (e) Venn diagram of the tBLASTn-identified *Arabidopsis* germplasm development-related homologues and key trend genes in Supplementary Table S3.

We categorized the DEGs across various developmental stages and three experimental lines into 20 distinct modules based on weighted gene co-expression network analysis (WGCNA) (Fig. 5a and Supplementary Table S4). We selected three traits – stage, line and fertility (fertility status) – to perform a module-trait correlation analysis (Fig. 5b and Supplementary Table S5). The results showed that the three different developmental periods (stages) were strongly negatively correlated with the brown module (*r* = −0.87, *P* = 3 × 10^9^) and strongly positively correlated with the royal blue module (*r* = 0.86, *P* = 7 × 10^9^) (Fig. 5b and Supplementary Table S5). Different lines (Line) displayed a strong positive correlation with the turquoise module (*r* = 0.82, *P* = 1 × 10^7^) and a strong negative correlation with the dark green module (*r* = −0.96, *P* = 1 × 10^14^) (Fig. 5b and Supplementary Table S5). Fertility showed a strong positive correlation with the turquoise module (*r* = 0.97, *P* = 7 × 10^17^) and a strong negative correlation with the dark green (*r* = −0.7, *P* = 5 × 10^5^) and salmon (*r* = −0.71, *P* = 3 × 10^5^) modules (Fig. 5b and Supplementary Table S5).

Fisher’s test was applied to integrate WGCNA and trend analysis results and to examine the overlap between genes showing significant trends and those identified in the WGCNA profiles. This revealed significant enrichment in the dark magenta and saddle-brown modules with *P* < .05 and odds ratio > 1 (Supplementary Table S6). The genes in the dark magenta module were mainly enriched in pathways such as phenylpropanoid biosynthesis; flavonoid biosynthesis; stilbenoid, diarylheptanoid and gingerol biosynthesis; ubiquinone and other terpenoid-quinone biosynthesis; starch and sucrose metabolism; other glycan degradation; phenylalanine metabolism; flavone and flavonol biosynthesis; sphingolipid metabolism; anthocyanin biosynthesis; and phenylalanine, tyrosine and tryptophan biosynthesis (Fig. 5c). The genes in the saddle-brown module were mainly enriched in pathways such as the biosynthesis of unsaturated fatty acids, zeatin biosynthesis, starch and sucrose metabolism, plant MAPK signalling pathway and plant hormone signal transduction (Fig. 5d). Notably, the shared enriched pathways between this module and those exhibiting significant trends were plant hormone signal transduction pathways (Figs. 4d and 5d).

**Figure 6:**
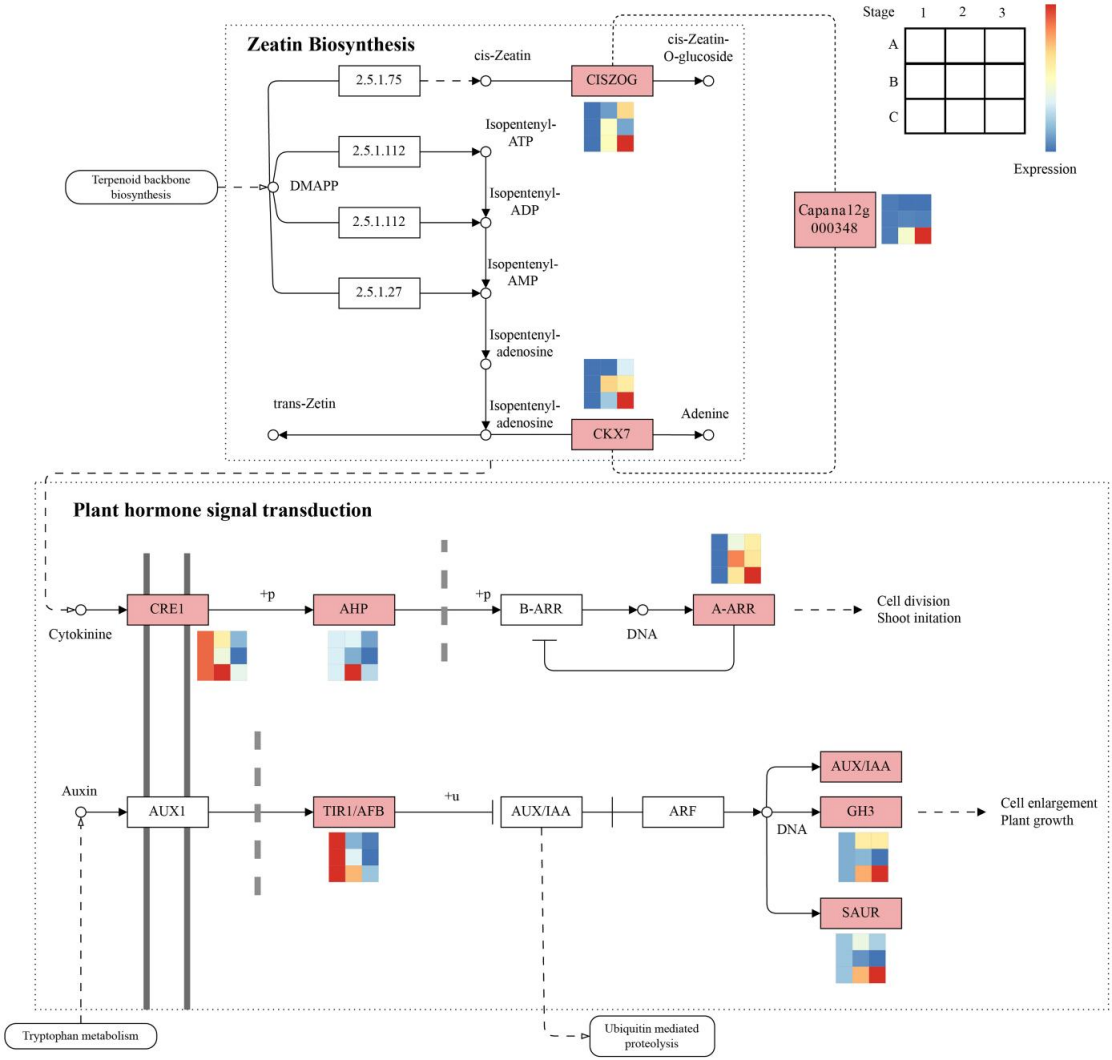
The expression changes of key genes involved in pathways associated with plant hormone-signal transduction and the zeatin biosynthesis pathway. Rounded rectangles and squares represent the pathways and gene names, respectively. Squares with a red background indicate DEGs. In the heatmap, each row corresponds to a different line, and each column represents a distinct stage.

We identified 12 high-confidence candidate genes potentially involved in chilli pepper germplasm development by intersecting tBLASTn-identified *Arabidopsis* germplasm development-related homologs (see Materials and Methods) with key trending genes (Supplementary Table S3) (Fig. 5e). Among the 12 candidates, *Capana02g001716* (receptor kinase-like protein Xa21) and *Capana12g000348* (probable leucine-rich repeat receptor-like protein kinase) were found in the saddle-brown module and were significantly enriched in the zeatin biosynthesis pathway (Fig. 5d). Zeatin biosynthesis is a critical upstream component of the plant hormone signal transduction networks (Fig. 6).

We focused on the expression patterns of genes involved in pathways related to zeatin biosynthesis and plant hormone signal transduction. Specifically, the small auxin upregulated RNA (*SAUR*), two-component response regulator (*A-ARR*) and auxin-responsive protein (*GH3*) genes in the plant hormone signal transduction pathway and *Capana12g000348*, cytokinin dehydrogenase 7 (*CKX7*) and *cis*-zeatin O-glucosyltransferase (*CISZOG*) genes in the zeatin biosynthesis pathway were gradually upregulated across developmental stages in the restorer line (Fig. 6). The auxin signalling F-box (*TIR1/AFB*) gene showed a pattern of gradual downregulation across developmental stages in the restorer line (Fig. 6). Additionally, the Cytokinin Response 1 (*CRE1*) and histidine-containing phosphotransfer factor (*AHP*) genes in the plant hormone signal transduction pathway displayed initial upregulation followed by downregulation (Fig. 6). Changes in the expression of key genes associated with plant hormone signal transduction pathways, including receptor kinase protein (*Capana12g000348*), *CISZOG*, *CKX7*, *CRE1*, *AHP*, *A-ARR*, *TIR1*, *GH3* and *SAUR* were further validated using qRT-PCR and were consistent with the transcriptomic trends across developmental stages (Supplementary Figure S3). A strong positive correlation was observed between the RNA-Seq data and the qRT-PCR validation results of these genes, with Pearson correlation coefficients ≥ 0.84 (Supplementary Figure S3).

## Discussion

CMS is a common biological phenomenon observed during the production of hybrid chilli pepper varieties. Transcriptomic analyses were conducted at different stages in the sterile, maintainer and restorer lines. Pentose and glucuronate interconversion pathways were particularly enriched in most comparison groups (Fig. 3). Sugar metabolism plays a pivotal role in plant development and is tightly coupled with sugar-signalling pathways (Tian et al. 2021). This coupling is achieved through the production of sugar-signalling molecules such as pentose, glucuronate and sucrose or by signalling within the metabolic process itself (O’Hara et al. 2013). Sugar metabolism disorders can adversely affect pollen and eventually lead to male sterility (Datta et al. 2002). Plant fertility is closely linked to pollen development, maturation and germination (Chao et al. 2016). In the present study, the pentose and glucuronate interconversion pathways were involved in the construction of a regulatory network of CMS in chilli pepper. Consistent with our results, the sugar metabolism signalling pathway in the seeds of the Chinese chestnut (*Castanea mollissima*) is involved in regulating male sterility (Zhang et al. 2015). Similarly, comparative transcriptome analysis revealed blocks in sugar metabolism in *Brassica napus* L. male sterility (Li et al. 2015). Additionally, sucrose metabolism and accumulation are altered during the development of T-type CMS in wheat (*Triticum aestivum*) (Ba et al. 2019). In contrast, pentose and glucuronate interconversion pathways participate in the fertility stability of cotton CMS-D2 restorer lines under heat stress (Wang et al. 2024).

Plant processes such as development, fertility maintenance and stress resistance require chemical energy, with development being particularly energy-intensive (Liu et al. 2020). Plant mitochondria play a critical role in photosynthesis as they catalyse the biosynthesis of carbon skeletons necessary for carbon fixation and several cofactors (Zancani et al. 2020). Impairment of ATP synthesis is frequently observed in CMS plants. Although such a metabolic deficiency may be sustained in numerous vegetative tissues, it invariably results in abortive pollen formation owing to the substantial energy demands inherent to developmental progression (Carlsson et al. 2008). As expected, mitochondrial energy deficiency leads to male sterility in specific CMS systems (Horn et al. 2014). However, photosynthesis produces large amounts of adenosine triphosphate (ATP). Multiparametric real-time sensing of cytosolic physiology links hypoxia responded to mitochondrial electron transport. These findings were important for understanding mitochondrial energy metabolism and oxygen production (Wagner et al. 2019). The redox status is an important determinant of germ cell development at the presporogenous stage and is involved in regulating plant sterility (Linde and Walbot 2019). We hypothesized that photosynthesis influences the status of the pre-porogenous developmental stage in chilli pepper flower buds through ATP and oxygen synthesis.

A vast majority of secretory and membrane proteins are processed in the endoplasmic reticulum. Defective protein processing and decreased levels of total free amino acids would repress the biosynthesis of proteins with different functions through various pathways (Ning et al. 2018). The development of the chilli pepper pollen involves the synthesis of numerous proteins (Fang et al. 2016). This result is consistent with our findings (Fig. 5d). Among the fertile, maintainer and restorer lines, DEGs were enriched in protein processing in the endoplasmic reticulum, which is likely an important factor in regulating male sterility in chilli peppers. Most of the differentially expressed proteins in chilli pepper pollen development are transferases, which may play important roles in protein processing such as acetylation, amination and glycosylation during another development (Fang et al. 2016).

In our study, WGCNA showed that *Capana12g000348* (a probable leucine-rich repeat receptor-like protein kinase) may affect cytokinin synthesis by regulating the expression of genes in the zeatin biosynthesis pathway, such as cytokinin dehydrogenase 7 (*CKX7*) and *cis*-zeatin O-glucosyltransferase (*CISZOG*), ultimately affecting cytokinin synthesis. Leucine-rich repeat receptor-like protein kinases (LRR-RKs) belong to the largest family of plant receptor kinases. LRR-RKs play crucial roles in plant development by regulating cell division, organ morphology and inflorescence composition (Li et al. 2024). Consistent with our results, zeatin biosynthesis is involved in regulating plant development through LRR-RK activation in *Arabidopsis thaliana* (Uchida et al. 2011). The cytokinin biosynthesis signalling pathway involves cytokinin dehydrogenase and *cis*-zeatin O-glucosyltransferase, which serve as transcriptional regulators. Cytokinins are essential plant hormones that regulate cell division, shoot meristem initiation, leaf and root differentiation, vascular patterning, fertility and seed development (Müller and Sheen 2007). A metabolomic study revealed that cytokinins participate in regulating CMS in Chinese cabbage (Yang et al. 2023). Cytokinins play a critical role in reproductive development in rice (Nongpiur et al. 2024). Cytokinins are candidates for engineering male fertility in *Sorghum* (Dhaka et al. 2020). Screening of DEGs and localization analysis of female gametophytes in *Pinus tabuliformis* Carr showed that cytokinin-related signalling pathways were blocked in sterile ovules (Gong et al. 2022).

In plant hormone signal transduction, *SAUR*, *A-ARR* and *GH3* genes showed gradually upregulated expression patterns across the developmental stages in the restorer line (Fig. 6). qRT-PCR experiments validated these results (Supplementary Figure S3). *SAUR* is rapidly activated in response to auxin hormones and significantly affects plant growth and development (Huang et al. 2023). In our study, *SAUR* showed a gradually upregulated expression pattern in the restorer line, which could be related to cell wall acidification (Stortenbeker and Bemer 2019). *SAUR* expression is significantly upregulated during the lateral root meristem stage in the model organism *Arabidopsis thaliana* following ACC treatment (Markakis et al. 2013). The increased *A-ARR* expression levels observed in our study might be related to stem development, which has been confirmed in Chinese kale (*Brassica oleracea var. alboglabra* Bailey) (Zheng et al. 2022). The *GH3* gene encodes acyl acid amidosynthetases, many of which have been shown to modulate the levels of active plant hormones or their precursors. The anther is part of the stamen, a male reproductive organ in plants, and *GH3* participates in regulating its development (Jeong et al. 2021). This finding is consistent with our research findings.

Auxin signalling F-box (*TIR1/AFB*), Cytokinin Response 1 (*CRE1*) and histidine-containing phosphotransfer factor (*AHP*) participate in CMS fertility regulation in chilli peppers (Fig. 6, Supplementary Figure S3). In our study, *TIR1/AFB* showed gradual downregulation across developmental stages in the restorer line, possibly influenced by ubiquitination (Qi et al. 2022). AFB auxin receptors are F-box subunits of ubiquitin ligase complexes. In response to auxins, they associate with Aux/IAA transcriptional repressors and target them for degradation via ubiquitination (Parry and Estelle 2006). It has been reported that *CRE1* maintained energy supply and intracellular metabolic stability under cold stress in chilli peppers (Song et al. 2024). In our study, *CRE1* was identified as a CMS fertility regulator, which may be due to its involvement in regulating plant cell division and differentiation (Inoue et al. 2001). The histidine-containing phosphotransfer factor (*AHP*) displayed initial upregulation, followed by downregulation. This may be related to the cytokinin signalling pathway (Keshishian and Rashotte 2015). Cytokinin is an essential plant hormone involved in a wide range of plant growth and developmental processes and is controlled through its signalling pathways (Jung et al. 2008). The cytokinin signalling pathway involves the coordination of three types of proteins: histidine kinase receptors to perceive the signal, histidine phosphotransfer proteins to relay the signal, and response regulators to provide signal output (Bertheau et al. 2012).

## Conclusions

Male sterility is an efficient approach for the commercial exploitation of heterosis. To date, no regulatory network for chilli pepper fertility genes has been constructed. We compared the transcriptomes of flower buds of chilli peppers in the sterile, maintainer and restorer lines to address this knowledge gap. Differential gene expression, trends and weighted gene co-expression network analyses were performed to construct a gene regulatory network for CMS in chilli pepper. *SAUR*, *A-ARR*, *GH3, Capana12g000348*, *CKX7*, *CISZOG*, *CRE1, AHP* and *TIR1* were identified as the regulators of CMS fertility in chilli pepper. Altogether, our results increase our understanding of the candidate genes and pathways associated with male sterility and may be used to explore the biological processes and molecular mechanisms underlying CMS in chilli peppers.

## Supplementary Material

plaf070_Supplementary_Data

## Data Availability

The datasets generated and analysed during the current study are available in the NCBI SRA repository (http://www.ncbi.nlm.nih.gov/bioproject/PRJNA1175070).
