# Supplementary material for: Comparative transcriptome analysis provides insights into the gene regulation network of cytoplasmic male sterility in chilli pepper
Source: AoB Plants. 2026 Jan 7;18(1):plaf070. doi: 10.1093/aobpla/plaf070 (PMC12778331; doi:10.1093/aobpla/plaf070)
Supplement: plaf070_Supplementary_Data [file plaf070_supplementary_data.zip › supporting information.pdf]

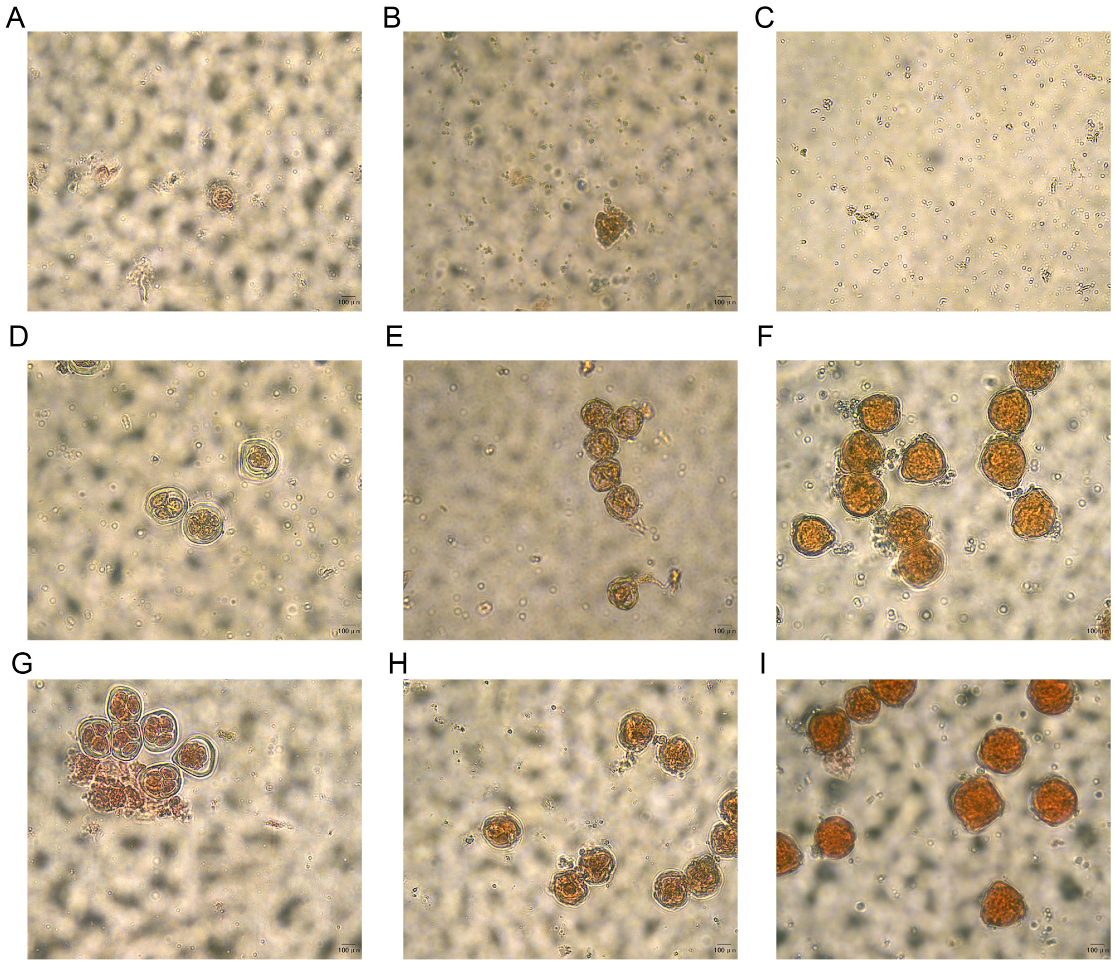

Figure S1. Comparative micro-morphology of pollen development in sterile line 014A (A-C), maintainer line 014B (D-F), and restorer line 014C (G-I) across three stages including Stage 1 (A, D, and G), 2 (B, E, and H), and 3 (C, F, and I).

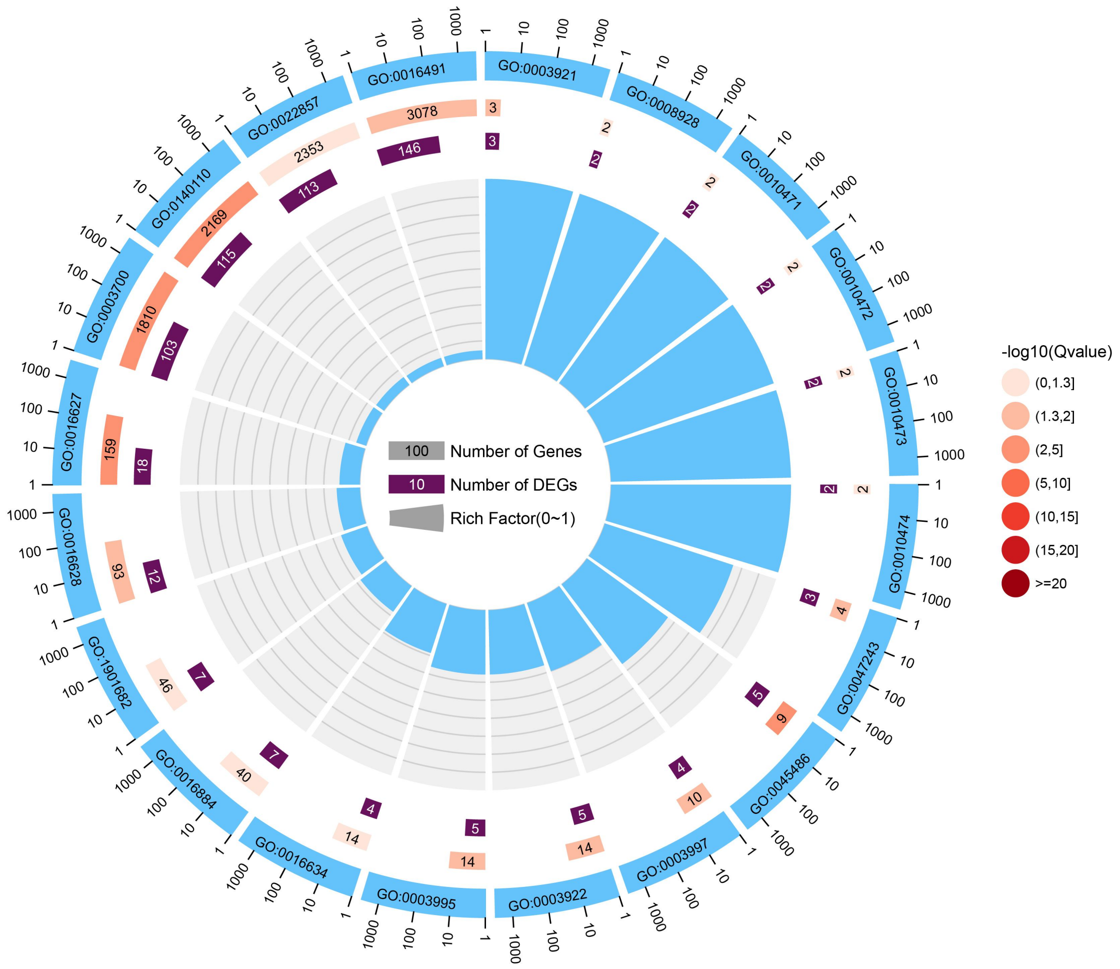

Figure S2. GO enrichment analysis for genes with similar trends between Trend_A and Trend_B, but different from Trend_C.

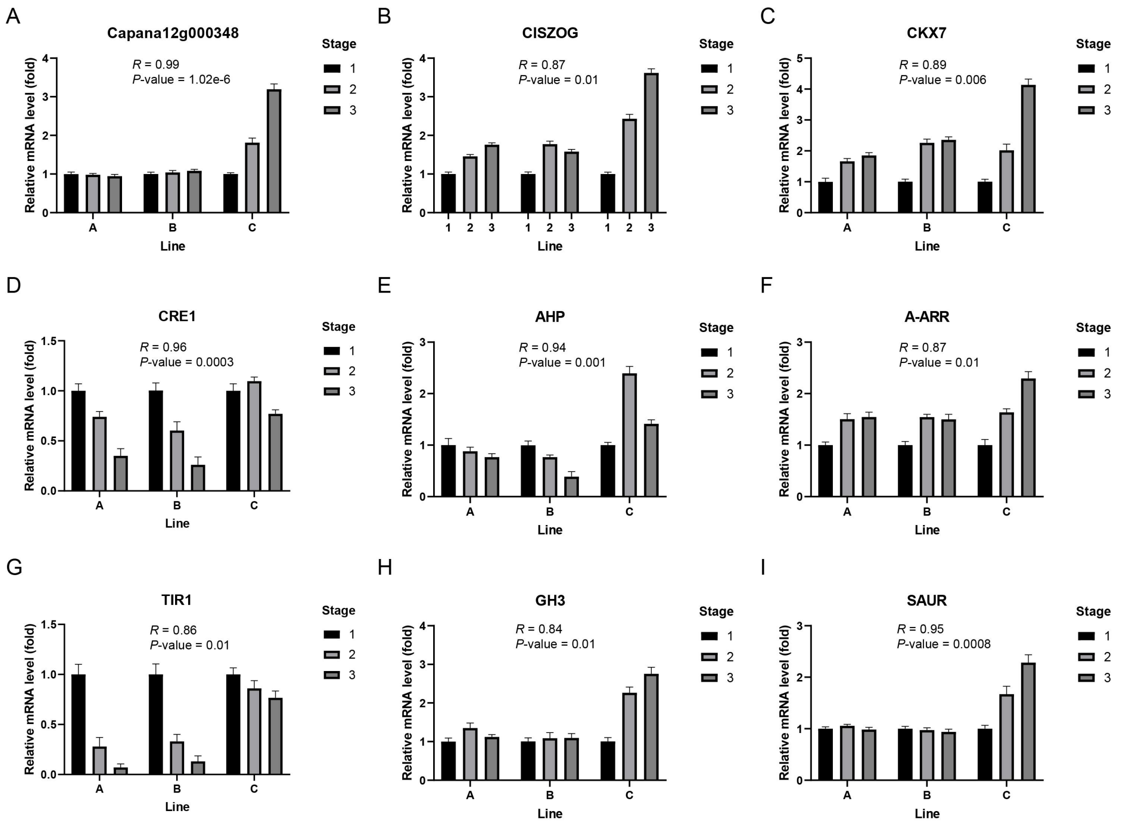

Figure S3. The qRT-PCR results of the key genes associated with plant hormone-signal transduction pathway including *Capana12g000348* (A), *CISZOG* (B), *CKX7* (C), *CRE1* (D), *AHP* (E), *A-ARR* (F), *TIR1* (G), *GH3* (H), and *SAUR* (I). The text annotations in each figure indicate the Pearson correlation coefficient (*R*) and the corresponding *P*-value between the RNA-Seq and qRT-PCR results.
